# Domain-specific AI segmentation of IMPDH2 rod/ring structures in mouse embryonic stem cells

**DOI:** 10.1186/s12915-025-02226-7

**Published:** 2025-05-12

**Authors:** Samuel T. M. Ball, Meagan J. Hennessy, Yuhan Tan, Kai F. Hoettges, Neil D. Perkins, David J. Wilkinson, Michael R. H. White, Yalin Zheng, David A. Turner

**Affiliations:** 1 Institute of Life-Course and Medical Sciences, Faculty of Health and Life Sciences, William Henry Duncan Building, University of Liverpool, Liverpool, UK; 2 Department of Electrical and Electronic Engineering, Faculty of Science and Engineering, University of Liverpool, Liverpool, UK; 3 Faculty of Medical Sciences, Biosciences Institute, University of Newcastle, Newcastle, UK; 4 Faculty of Biology, Medicine and Health, School of Biological Sciences, University of Manchester, Manchester, UK

**Keywords:** IMPDH2, Rods and rings, Loukoumasomes, Cytoophidia, Nematosomes, Embryonic stem cells, AI segmentation, Deep learning, Image processing, Convolutional neural networks

## Abstract

**Background:**

Inosine monophosphate dehydrogenase 2 (IMPDH2) is an enzyme that catalyses the rate-limiting step of guanine nucleotides. In mouse embryonic stem cells (ESCs), IMPDH2 forms large multi-protein complexes known as rod-ring (RR) structures that dissociate when ESCs differentiate. Manual analysis of RR structures from confocal microscopy images, although possible, is not feasible on a large scale due to the quantity of RR structures present in each field of view. To address this analysis bottleneck, we have created a fully automatic RR image classification pipeline to segment, characterise and measure feature distributions of these structures in ESCs.

**Results:**

We find that this model can automatically segment images with a Dice score of over 80% for both rods and rings for in-domain images compared to expert annotation, with a slight drop to 70% for datasets out of domain. Important feature measurements derived from these segmentations show high agreement with the measurements derived from expert annotation, achieving an *R*^2^ score of over 90% for counting the number of RRs over the dataset.

**Conclusions:**

We have established for the first time a quantitative baseline for RR distribution in pluripotent ESCs and have made a pipeline available for training to be applied to other models in which RR remain an open topic of study.

**Supplementary Information:**

The online version contains supplementary material available at 10.1186/s12915-025-02226-7.

## Background

### Introduction

Multiparameter fluorescence microscopy is a powerful and versatile tool in modern biology which allows both qualitative and quantitative measurement of biological processes in fixed or live samples [1]. Despite the rapid expanse in both the availability and technological innovation seen in the field of quantitative microscopy, adequately analysing the data generated from the microscope remains a major bottleneck [2]. The recent advances in the application of artificial intelligence (AI) stand as a useful yet underutilised tool to address this issue.

**Fig. 1:**
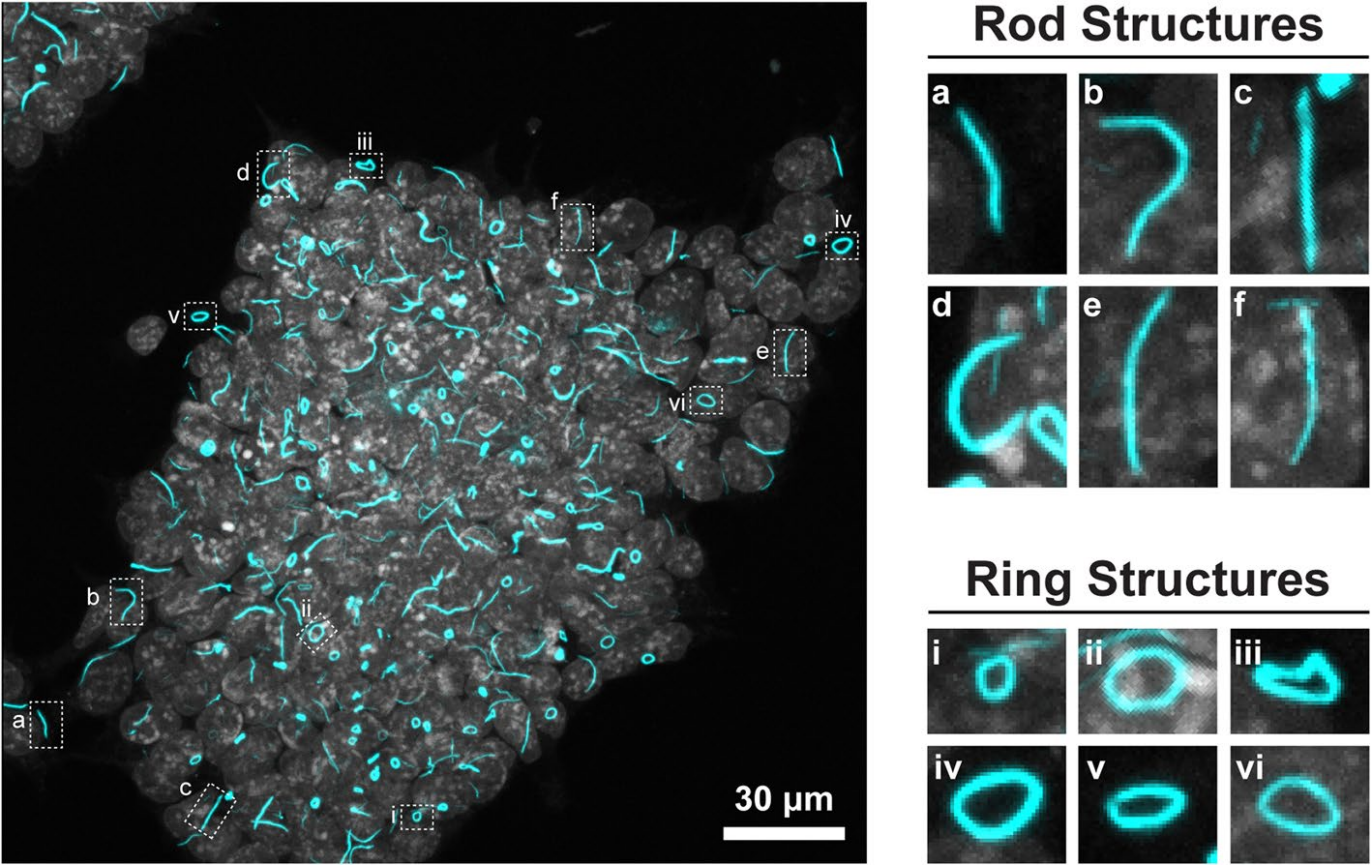
IMPDH2 rod-ring structures in pluripotent mouse embryonic stem cells. A representative maximum intensity projection of fixed mouse embryonic stem cells (left) showing DAPI (grey) which marks the nuclei, and IMPDH2 (cyan). Magnified examples of rods (top right; inserts a–f) and rings (bottom right; inserts i–vi) are shown and are indicated by hashed boxes in the larger figure. The number of rods-rings in this image is in excess of 200. Scale bar indicates 30 μm

One potential application for deep learning techniques can be seen in the study of inosine monophosphate dehydrogenase 2 (IMPDH2), the enzyme that catalyses the rate-limiting step in the synthesis of guanine nucleotides, and is a critical component of cell homeostasis and metabolism [3]. IMPDH2 can exist in two major conformational classes. The first is in a dissociated form where IMPDH2 is distributed homogenously throughout the cytoplasm [4]. The second is in large macromolecular complexes resembling rod or ring (RR) structures (Fig. 1) [5, 6]. These RR structures have been seen across a variety of models including but not limited to fission yeast [5, 7], mouse embryonic stem cells (ESCs), *Danio rerio* larvae (8 days post coitum) [8], *Drosophila melanogaster* egg chambers [8], in the adult sympathetic neurons in rats [8, 9], and in a variety of human cancer cell lines [5]. While often including the IMPDH2 enzyme, RRs can exist independent of IMPDH2 and/or include other metabolic enzymes such as cytidine triphosphate synthase 1 (CTPS1) [5, 7], glutamate synthase [8] and GDP-mannose pyrophosphorylase [8]. Although this has led to the suggestion that higher-order molecular conformation may be important for fine-tuning enzymatic kinetics [8, 9], to date the field has lacked the tools to systematically classify and quantify RR structures independently. Without basic quantification tools to track rods and rings in the cell, the biological role of RR conformation remains an open question.

Mouse ESCs are a useful model system to study the regulation of these structures, as the IMPDH2 conformation differs depending on their state (Fig. 1 and [5, 6]). When ESCs are maintained in pluripotent conditions, IMPDH2 is mostly in the rod-ring conformation. During differentiation, however, these structures break down and IMPDH2 becomes more homogenously cytoplasmic. As these structures have a clear morphology, single instances of rods and rings are relatively straightforward to analyse, and manual quantification similar to our previously published protocols [10, 11] or simple thresholding using, for example, ImageJ/FIJI, is possible [12–14]. However, within a single field of view, there can be dozens to over a hundred rods and rings, rendering manual quantification impractical, especially if one is to analyse enough structures for meaningful statistical analysis. Furthermore, it is necessary for analysis methods to be able to distinguish between these two broad classifications (rod or ring) to examine whether these conformations have distinct biological functions.

#### Software requirements

Due to the number of these structures within each image (Fig. 1) and the impractical nature of current analysis methods, it is clear that any software that could tackle our analysis would need to:

1. Identify rods or rings with a high degree of accuracy, consistently over multiple experimental replicates, conditions, and fluorescence intensities compared with manual identification;
2. Be automated, requiring little to no adjustments or corrections;
3. Run through datasets at least an order of magnitude quicker than manual analysis;
4. Be user-friendly, and able to export the data in a format that can be interrogated by third-party graphing/analysis packages.

We therefore set out to develop a deep learning approach to establish a numerical baseline for IMPDH2 rod and ring distribution in mice. Since these RR structures are present in multiple contexts (e.g. IMPDH2 [5], CTPS1 cytoophidia [5, 7], loukoumasomes [15]), the solution we develop is expected to be of use to multiple groups for the quantitative analysis of similar cellular structures.

#### Related work and background theory

Image segmentation is a commonly investigated computer vision task, with models for object detection via bounding boxes [16], prompt-based segmentation [17], semantic segmentation [18], instance segmentation, and panoptic segmentation [19]. The methodology for using these models is typically split into two cases: in the first, pretrained models are used directly on new datasets to detect objects or segment images. In the second, these model architectures are partially or fully retrained on the new data to achieve greater results.

Commonly used applied models such as SegFormer [18] come with labels for general use (e.g. dog, person, train), but are inappropriate for RR segmentation as there is no “rod” or “ring” output in the pretrained model outputs. The large size of these models, combined with the relatively small size of some microscopy imaging datasets typically results in these larger architectures being outperformed by simpler models at a smaller scale [20]. Other models, such as Segment Anything [17] or Cellpose [21] take a label-free approach to segmentation, using user prompting to infer structure in the image. While these models can increase the speed of segmentation and annotation for many biomedical tasks, they cannot achieve the end-to-end segmentation like those above. Therefore, the current analytical resources available to the field leave segmenting and classifying RRs in a “grey area” that can only be addressed by training a model which is tailor-made to address this classification problem.

Training bespoke, application-specific models allow for specific considerations to be made for the type of data being analysed, such as preprocessing or model design. By training a model on a specific dataset, higher performance is usually achieved compared to generalised models due to a smaller domain space; however, performance out-of-domain (i.e. on data sufficiently dissimilar to the training data) is typically much worse.

## Implementation

### AI methods

#### Training

**Fig. 2:**
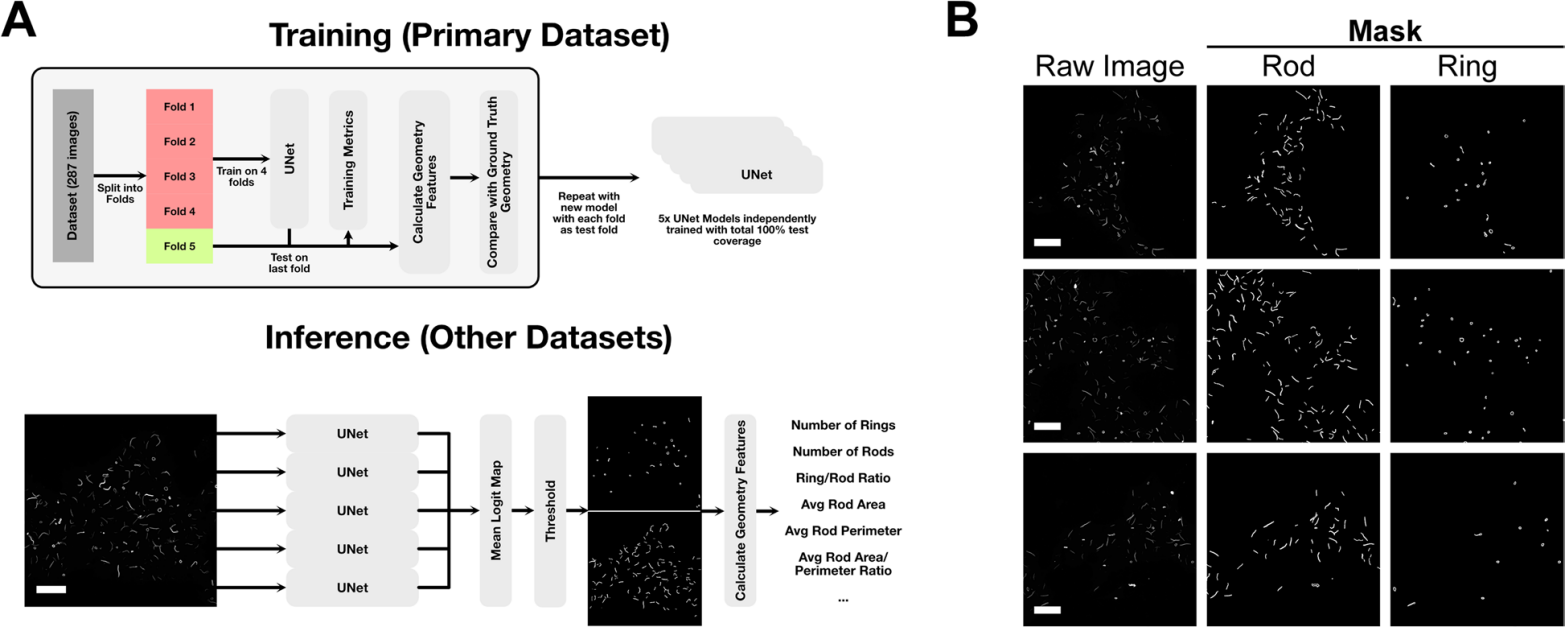
Training and inference protocol, and example training labels for RR segmentation. **A** Training and inference protocol used in this work. The training datasets were split into 5 disjoint sets, with a UNet model trained on each combination of 4 sets, testing on the final test set each time. After the training and testing on the primary dataset is complete, this gives us 5 UNet models that are used in ensemble for inference, with the output logits being averaged and thresholded to gain the final segmentation. **B**  A sample of the raw images (left) along with the expert annotations for Rods and Rings used for training. It is worth noting that although these labels are provided by a highly skilled expert in the field, they are by no means an “absolute” ground truth; and contain some implicit biases and variations between the images. Scale bars represent 30 μm

For training the microscopy AI models, we used 5 UNet models [22] in an ensemble model (Fig. 2A). Each input image is scaled to 512 × 512 pixels, and the output logits of the UNet models are averaged to give an aggregate logit map of the output. For downstream binary classification tasks, the output was thresholded at a value of 0.2, chosen due to the ensemble model’s propensity to over-classify negative classes (i.e. empty space) in the image. The dataset totalled 287 training images, each with an accompanying annotation mask for RRs (Fig. 2B). This was split into 5 disjoint folds, where each model was trained on 4 of these folds and tested on the last one, with each model testing on a different fold. This ensured complete testing coverage of the entire dataset, and repeated training to ensure reliability of the protocol. Training was performed on a system with a NVIDIA GeForce RTX 3090 equipped using the Adam optimizer [16, 23] with an initial learning rate of 0.001, which decayed exponentially at a rate of 99.9% per epoch. During training, the loss for the training was recorded along with the loss on the testing dataset, although the latter was not used for hyperparameter optimisation to avoid overfitting and preserve the integrity of the experiment.

#### Post-processing

After the models were trained, the masks were used to construct polygons for the RRs using a simple contour fitting algorithm [24]. For each image, several measurements were taken, which were deemed biologically relevant (e.g. the number of RRs, the average perimeter and area RRs, the ratio of area to perimeter of RRs) to study the utility of the model in the biological context.

#### Testing

**Fig. 3:**
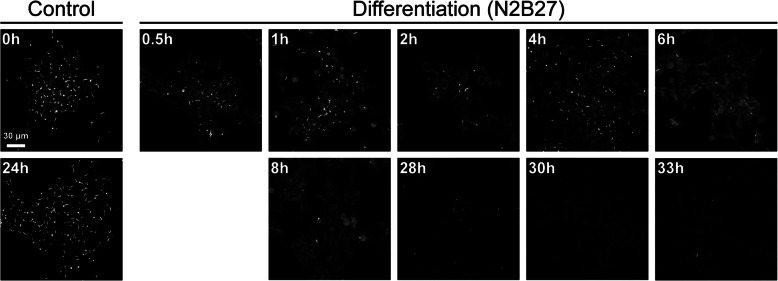
Differentiation of mouse ESCs and the loss of IMPDH2 RR structures. Examples of control cells at the start (0 h) at 24 h in pluripotency media (left), and cells over a period of 33 h, sampled at the indicated time-points following transition into differentiation media (N2B27). Scale bar indicates 30 μm

For testing the models on the original data, the final testing folds for each cross-validation set were predicted and compared against the ground truth binary masks provided by an expert annotator using both the Dice coefficient score and the per-pixel Jaccard score for pixel classification. In addition to the main dataset, we also tested the model on two further datasets: firstly, a time course dataset recorded with the same microscope as the primary dataset but in the context of an experiment examining the effect of differentiation media (N2B27) on the cell line. Here, the dataset is comprised of 306 image files at 11 different timepoints including two control points. In this case, the density of RRs quickly diminishes over time (Fig. 3). Testing the AI’s performance on this dataset not only tests its ability to segment images out of the training domain (i.e. images similar to those that it was trained on), but also serves as a simulation into how this model could be used in practice.

**Fig. 4:**
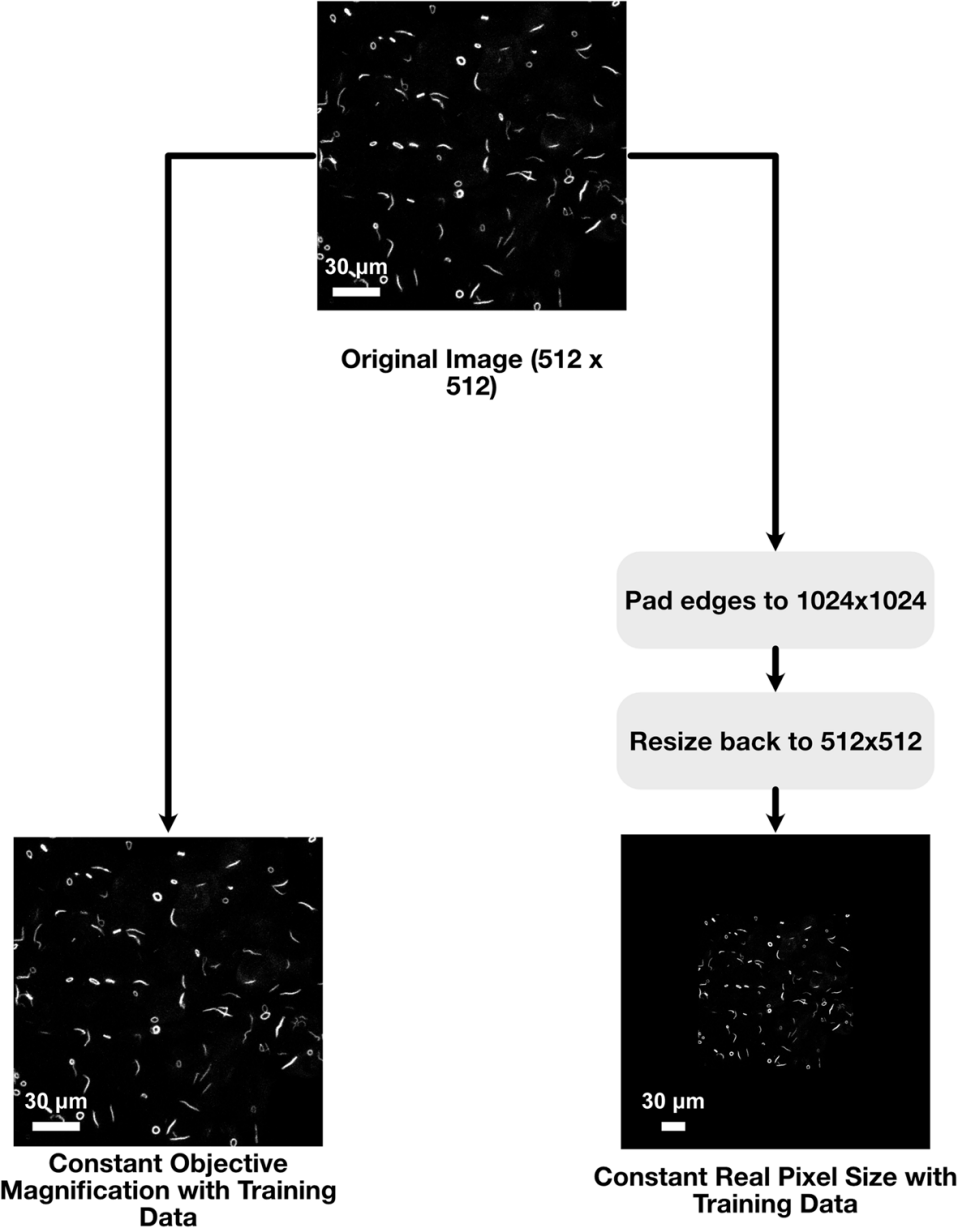
Preprocessing pipelines for datasets. In general, we found through development that very little preprocessing needed to be applied to the raw images before inputting into the model, with normalisation harming the segmentation results rather than helping. For the experiment with a change of microscope, two pipelines were developed due to a change in output resolution of the image: one fixing the objective magnification to that of the training data (e.g. scaled to the same pixel size with no cropping); and one fixing the real pixel size with that of the dataset (padding the image to the same size as the training image prior to scaling). We find that the latter far outperforms the former. Scale bars represent 30 μm

The final testing dataset examines the effect that distinct settings on a different confocal microscope would have on the AI’s performance. In this case, we have 90 images recorded on a different microscope from what was used on the primary dataset. This dataset tests multiple out-of-domain (i.e. unseen in training) factors, including image scaling and different microscopes that are previously unseen in the primary dataset. For this set, two preprocessing pipelines were used for comparison, as the new microscope’s resolution was different from the original. The first, where the objective magnification was kept constant with the primary microscope, and the second, where the image was cropped so the resolution of the cells (i.e. the average pixel width) was kept constant with the original microscope (Fig. 4).

For the time course and domain shift evaluations, since this data was not previously seen by the model; the ensemble model approach was used on the entire dataset, again recording the per-pixel Jaccard score and Dice coefficients per image. In both cases, the logit maps were also used to construct receiver operating characteristic (ROC) curves for each image, then averaged over the image set, giving a mean ROC curve along with a standard deviation range. Finally, for each model output, the summary metrics after post-processing were compared with those calculated from the ground truth; to test for overall utility of the model in a biological context. This was achieved by calculating the *R*^2^ score for each measurement to test for the strength of correlation of the measurements from the ground truth to the model output.

#### Image processing pipeline

Clear, in focus images from the Andor Dragonfly Microscope were exported as *.ims* files, and maximum intensity projections were created (1024 × 1024 pixels) before being converted to *.jpgs* for RR prediction to be made off from the model. For instances in which annotations were required, OpenCV [25] was used to draw initial contours off single channel maximum projection images. These were then manually cleaned and separated into rod and ring label layers in napari [26] which were exported as binary masks for training.

### Biological Methods

#### Routine mouse embryonic stem cell culture and differentiation

Wild-type E14-Tg2A mouse ESCs were seeded at a density of 1.2 × 10^4^ cells/cm^2^ on 0.1% (v/v) gelatin-coated flasks in ESL medium, which is comprised of GMEM (Gibco) supplemented with 15% foetal bovine serum (Gibco Cat. No. 10270–106), non-essential amino acids (Gibco), sodium pyruvate (Gibco), Glutamax™ (ThermoFisher, Cat. No. 35050038), β-mercaptoethanol (Gibco, Cat. No. 31350010; final concentration 0.05 mM), and LIF (QKine; QK018) in a humidified incubator maintained at 37 °C with 5% CO_2_. Cells were passaged every other day using TrypleE as a dissociation reagent. The culture medium was changed on non-passage days. For differentiation assays, cells were seeded directly in N2B27 [27, 28] and medium was changed each day [29]. N2B27 was kept at 4 °C protected from light and was used within 3 weeks. Cells were tested monthly and certified negative for mycoplasma.

#### Immunofluorescence

Cells were seeded at a density of 3.0 × 10^4^ cells/cm^2^ in 8-chambered Ibidi slides in ESL for 24 h [27, 28]. For samples that were gathered for the differentiation time course experiment, ESL media was exchanged for N2B27 at 24 h and cells were fixed at the defined intervals. Samples were then processed for immunofluorescence as previously described [10]. Prior to fixing, cells were washed with phosphate buffered saline without calcium and magnesium (PBS^−/−^). Single time point experiments were fixed for 30 min at room temperature with 4% formaldehyde (Thermo Scientific/Pierce). Samples that were part of the differentiation time course were fixed at the defined intervals with 4% formaldehyde for 10 min at 37 °C. After fixation, cells were washed multiple times (~ 10 min each) with PBS^−/−^ supplemented with 10% FBS and 0.2% Triton X100 (PBSFT), followed by 3-h-long washes in PBSFT to block and permeabilise the cells. Cells were then incubated with an antibody raised against IMPDH2 (12948-1-AP, Proteintech) diluted 1:2000 in PBSFT at 4 °C overnight. Cells were then rinsed three times with PBSFT and exposed to three lots of 1-h washes in PBSFT before incubation overnight with Alexa-488 conjugated secondary antibody diluted 1:500 in PBSFT with Hoechst (1:1000) to mark nuclei at 4 °C. Cells were then rinsed with PBS supplemented with 0.2% FBS and 0.2% Triton X100 (PBT) three times before 3-h-long PBT incubations at room temperature. After the final rinse, cells were mounted in ProLong Antifade (P36980, Thermo Fisher Scientific) and stored at 4 °C until imaging.

#### Confocal microscopy

Fixed and stained cells were primarily imaged on an Andor Dragonfly spinning disc confocal microscope mounted on an inverted Leica DMi8 base using a 40 × 1.4 NA oil-immersion objective. Hoechst and Alexa-647 were sequentially excited with 405 and 637 nm laser diodes respectively, and emitted light reflected through 450/50 nm and 700/25 nm bandpass filters respectively. An iXon 888 EM-CCD camera was used to collect emitted light, and data were captured using Fusion version 5.5 (Bitplane). For microscope comparison tests, samples were imaged on a Zeiss LSM800 Airyscan confocal microscope mounted on an inverted Axio Observer Z1 base using a Plan-Apochromat 63 × 1.40 NA Oil-immersion objective. Alexa-647 was excited with a 640-nm laser diode, with emitted light reflected through a variable secondary dichroic beamsplitter set between 656 and 700 nm. Emitted light was directed to a GaAsP photomultiplier tube, and data were captured using Zen Blue version 2.6. Each frame was a 1024 × 1024 pixel image (196.1 µm × 196.1 µm) where one pixel corresponded to ~ 0.192 µm. Z-stacks comprised a range between 62 to 158 steps, with each step size being 0.11 or 0.12 µm.

## Results

### Primary dataset

**Fig. 5:**
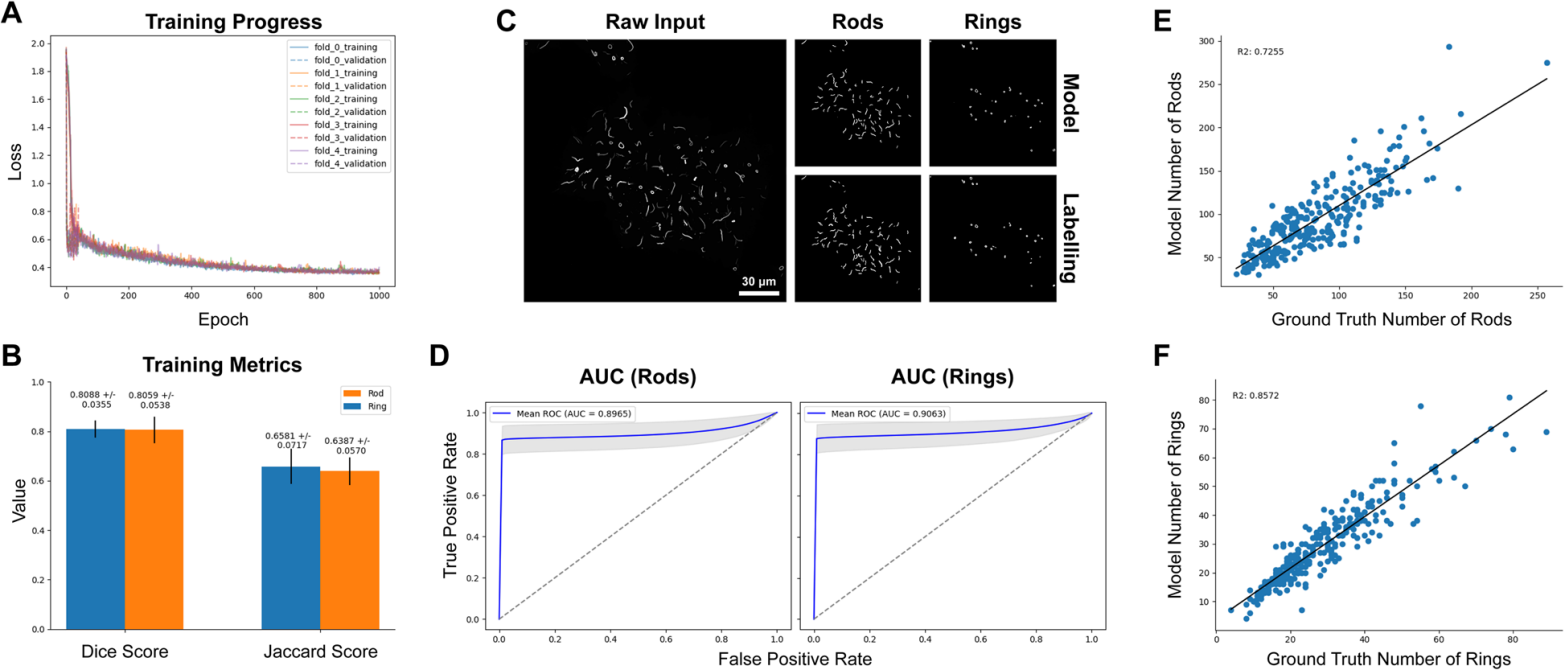
Model Training. **A** shows the training and testing losses of each of the models over time. We see that the models similarly converged over time, showing there were no training folds significantly different from others for training. **B** shows the mean Dice and Jaccard scores for the models tested on the holdout test set for the rod and ring mask recovery. **C** A representative example outputs along with the relative labels for the rods and ring masks. **D** Shows the per-pixel AUC curve for each of the masks. **E** and **F** show the correlation of the derived measurements from the model segmentations versus the segmentation provided by an expert annotator. A strong correlation here shows not only an accurate segmentation, but also a biologically helpful model that can be used for downstream analysis. Scale bar represents 30 μm

The model training progress shows convergence of the models on the training datasets while still not overfitting (Fig. 5A). After training, the models were tested on each testing fold using the Dice and Jaccard scores (Fig. 5B), and the logit maps were used to construct ROC curves (Fig. 5D). In both cases, we see high performance from the model, achieving an overall average Dice scores of 0.806 ± 0.054 and 0.809 ± 0.035 for rods and rings respectively and Jaccard scores of 0.639 ± 0.057 and 0.658 ± 0.072 for rods and rings, respectively. We also see a strong performance in the ROC curve analysis, achieving a mean AUC of 0.8965 and 0.9063 for rods and rings (Fig. 5D). In representative examples of model outputs (Fig. 5C), the model clearly segments the images well, apart from in a few select areas of disagreement that may be a result of annotation bias (the correlation of derived measurements in are also shown Fig. 5E, 5 and Additional file 1: Fig. S1). Overall, we see a strong correlation between the researcher derived measurements and those produced by the model for the number of rods and rings *R*^2^ of 0.7255 and 0.8572, but much lower for the other measurements (Additional file 1 Fig. S1), showing that the output of the AI can be used to track some biologically meaningful information within the domain of the dataset.

### Time Course Dataset

**Fig. 6:**
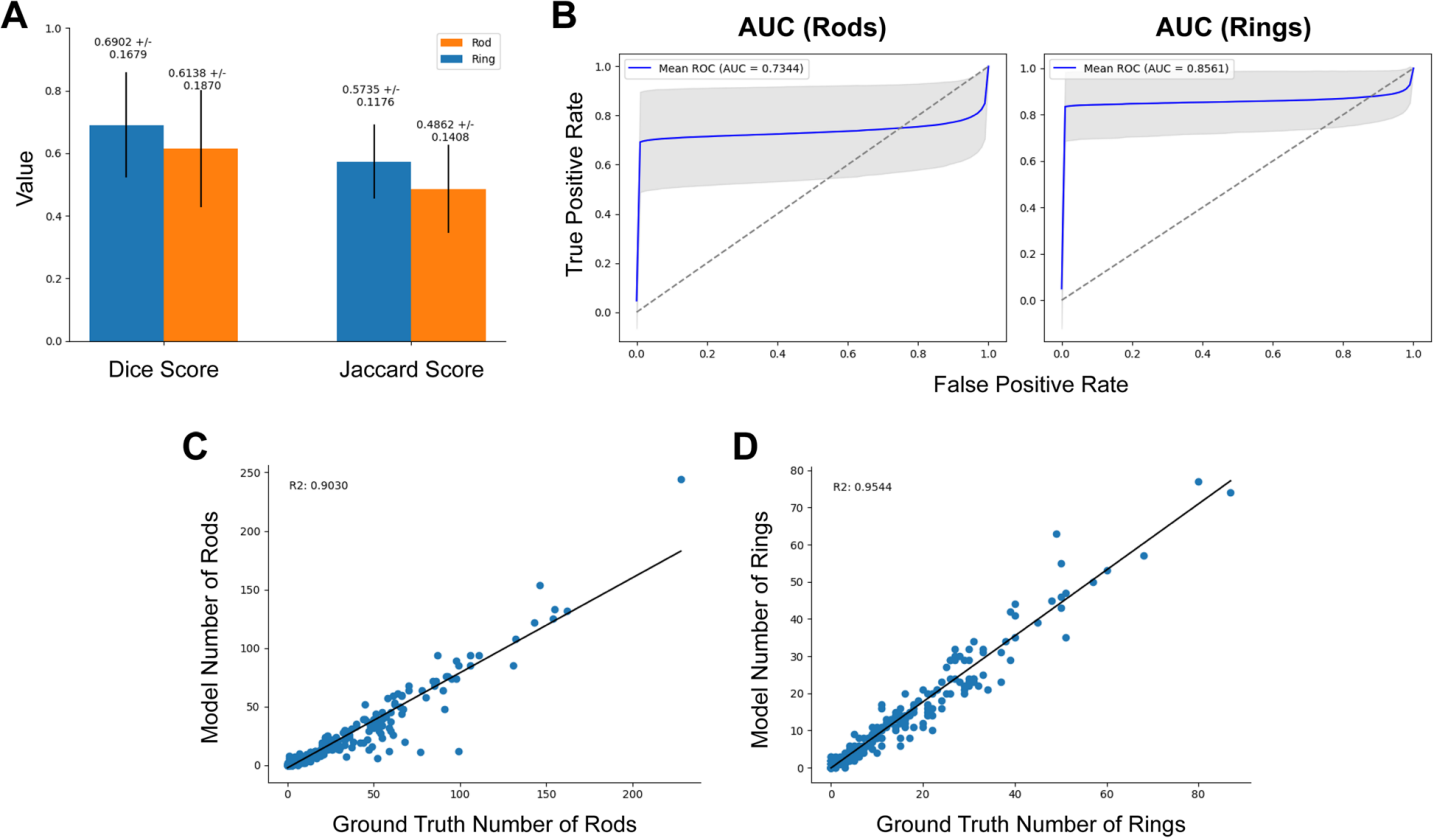
Time-course metrics. For the time-course dataset, the model trained on the primary dataset was applied via an ensemble mechanism with the resulting metrics to test for out-of-domain performance. **A** shows the Dice and Jaccard scores for rods and rings for each image compared with expert annotation. **B** shows the per-pixel AUC scores for both the rods and rings. **C** and **D** show the correlation between derived features from the segmentations for both the expert analysis and the model. As with the previous primary dataset, correlation here shows the model can recover meaningful biological information as well as pixel-wise accuracy

To test the model’s performance at predicting data outside of the training domain, an additional dataset was recorded that examined the effect of differentiation media on the RR structures over time, which permitted us to test the out of domain performance of the model. In this case, we again record the Dice and Jaccard scores (Fig. 6A) and show similar performance to the testing dataset albeit with some reduction in images with low numbers of RRs, with average dice scores of 0.637 ± 0.203 and 0.750 ± 0.194 for rods and rings respectively and Jaccard scores of 0.433 ± 0.201 and 0.441 ± 0.263 for rods and rings respectively. ROC curve analysis shows similarly high AUC scores for rods and rings compared to the primary dataset, with AUCs of 0.734 and 0.856 for rods and rings (Fig. 6B).

There is a high correlation between the ground truth expert-annotated masks and the output of the ensemble model, with *R*^2^ of 0.9030 and 0.9544 for rod and ring counts respectively (Fig. 6C, 6). This indicates that the model is robust to changes in rod ring morphology due to biological changes in the experimental conditions. The plots of several measurements split by their time point can be seen in (Additional File 2: Fig. S2). We show that the model is successfully able to find similar patterns in the time course experiment as the human annotations.

### Microscopy change dataset

**Fig. 7:**
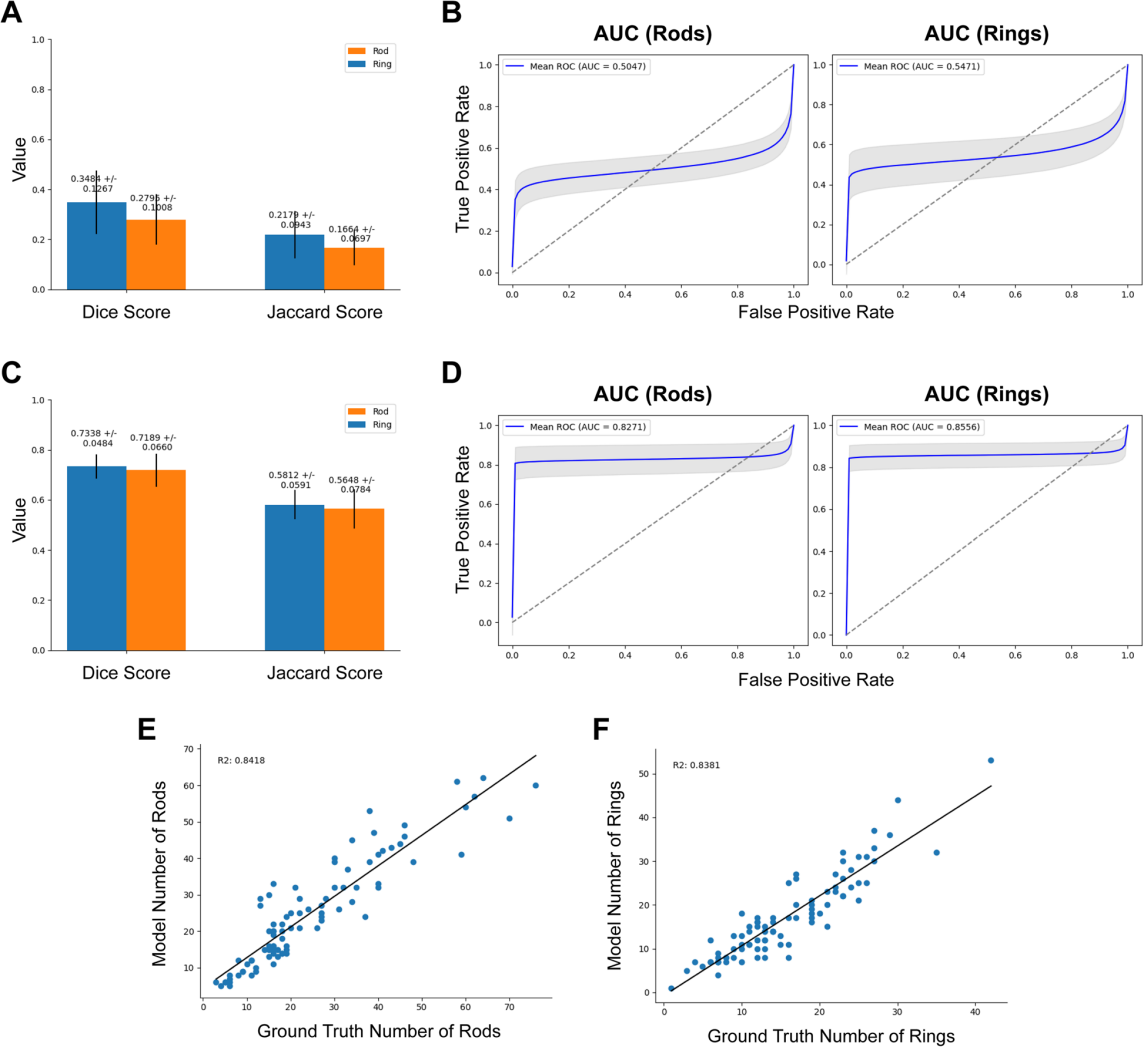
Microscopy experiment metrics. Data from a different microscope was used to test for application of the model out-of-domain. We find that when preprocessing fixes the objective magnification of the input data, the model performance is fairly poor. The Dice and Jaccard scores for rod and ring segmentation compared to expert analysis are shown (**A**), as well as the pixel-wise AUCs for rods and rings (**B**). By preprocessing the data to preserve the real pixel size compared to the primary dataset, we see much better model performance. **C** shows the Dice and Jaccard scores for the rod and ring segmentations after this preprocessing step and **D** shows the AUC scores. **E** and **F** show the correlation of the latter preprocessing pipeline model with the expert annotations. We again see a strong correlation with the expert annotation showing strong biological relevance of the model

The model was tested on a dataset recorded by a different microscope to examine the robustness of the model to changes in the nature of the images. These data were pre-processed in two ways as the microscope resolutions differ: one pipeline to keep the magnification constant with the primary dataset and another to keep the pixel height and width constant (Fig. 4). When examining the performance of the model, we see relatively low Dice scores of 0.2795 ± 0.101 and 0.3483 ± 0.127 and Jaccard scores of 0.166 ± 0.069 and 0.218 ± 0.094 for rods and rings, respectively (Fig. 7). Additionally, ROC analysis for the constant magnification dataset shows similarly disappointing results (Fig. 7B), with AUCs of 0.5047 and 0.5471 for rods and rings respectively. Representative examples qualitatively show that the model has unsuccessfully read these images as there are significant artefacts with the output masks (Additional File 3: Fig. S3).

Interestingly, for the pixel height and width constant preprocessing pipeline, we see much better results (Fig. 7C), with higher Dice scores of 0.719 ± 0.066 and 0.733 ± 0.048 and Jaccard scores of 0.565 ± 0.078 and 0.581 ± 0.059 for rods and rings respectively. Furthermore, in this case the ROC analysis shows much greater performance (Fig. 7D), with AUCs of 0.8271 and 0.8556 for rods and rings. Lastly, for the pixel height and width constant preprocessing pipelines, we also measure the correlation between expert-annotated mask measurements and the AI model output mask measurements (Fig. 7E, 7). We see a strong correlation between the two, with an *R*^2^ score of 0.8418 and 0.8381 for rod and rings counts.

### Development of a WebApp

Finally, throughout the development of this model we found it crucial to develop an accompanying webapp, allowing feedback from expert biologists in how to improve the model in the biological context. A screenshot of this model in practice is in Additional File 4: Fig. S4, and we have made the webapp code available for use by research teams in an easy-to-deploy manner [30].

## Discussion

In this work, we have developed a fully automatic end-to-end image segmentation pipeline for RR microscopy images that can derive biologically relevant measurements over the course of an experiment. However, we still feel like there are several open questions for this work:

### Comparison to existing methods

**Fig. 8:**
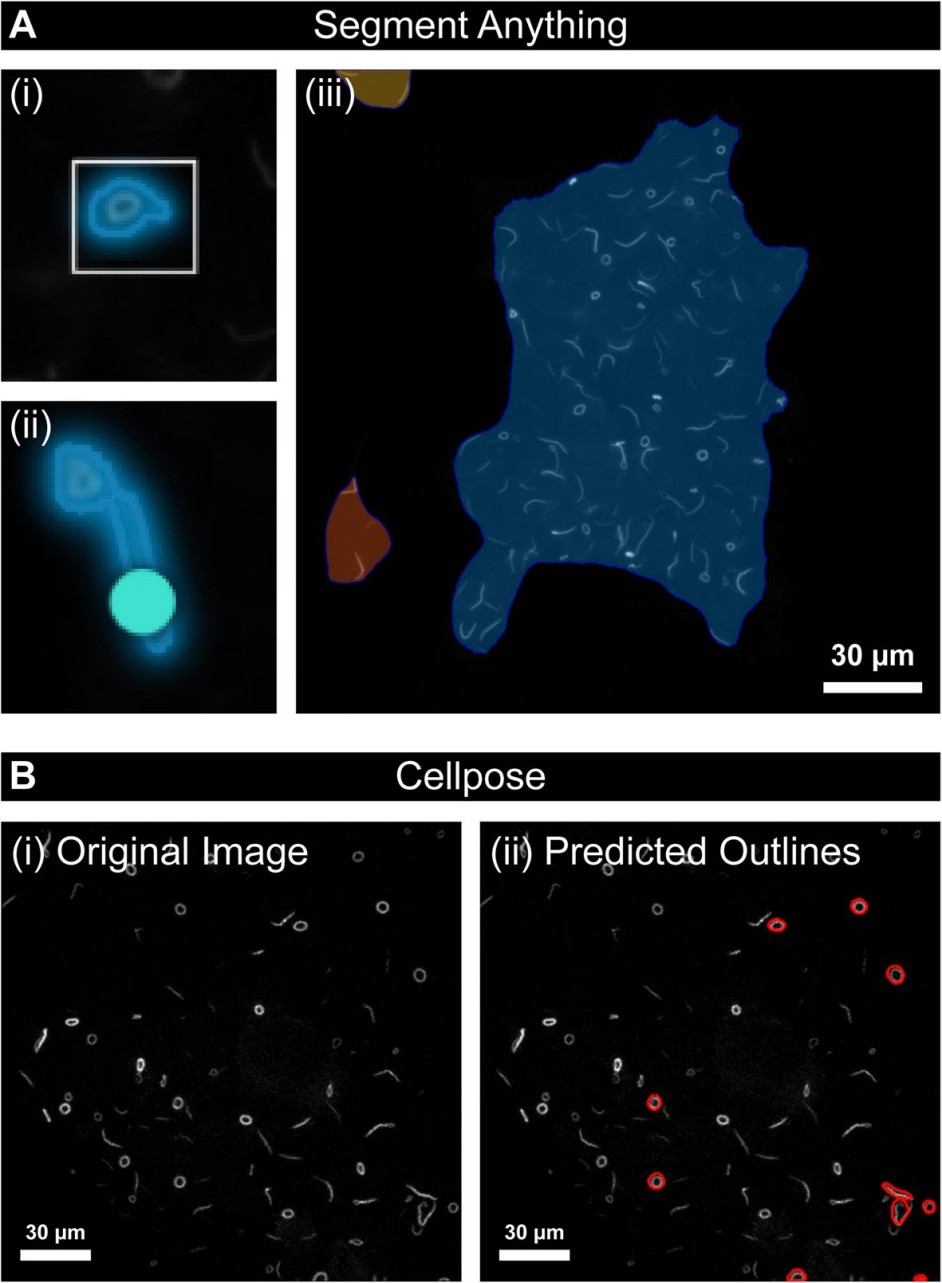
Example segmentations from Segment Anything and Cellpose. **A** Segment Anything provides context-free segmentations using user prompts to drive the segmentation process; however, we find that using the different modes for SAM, we get poor results for our specific datasets. (i) shows the segmentation using the box-prompt mode, and (ii) shows the segmentation using the point prompt mode. The representative example (ii) shows the model unable to differentiate between a rod and a ring object. (iii) shows the “Whole picture” approach, which fails to single out individual objects in the image. **B** The original image (i) and predicted outcomes (ii) using Cellpose show an inability to segment rods and rings correctly. Scale bars represent 30 μm

Pre-trained transformer-based models such as Segment Anything (SAM) [17] and Segformer [10] are all state-of-the-art models in their respective fields, however, all have significant drawbacks in this domain. SAM still requires some user input in detecting objects for segmentation, so the inclusion of the model alone in the pipeline would lose the automatic nature of the work. Since the throughput of microscopy can be high, this is crucial to the utility of the model. Furthermore, images segmented with SAM show significant errors in its segmentations (Fig. 8A). Cellpose is a similar solution, designed to be general to cellular applications, however, we find similar issues with the model’s ability to segment RRs (Fig. 8B). Similar issues exist for Segformer. This model segments without user input, but uses a pre-defined set of labels that do not include rods and rings. In development, retraining or fine-tuning these models was unsuccessful due to overfitting, perhaps due to the large size of these models compared with the relatively small size of our dataset.

### 3D versus 2D segmentation

The microscopy images effectively exist within three-dimensional space; the images used in this work are two-dimensional slices of these three-dimensional cells. As many two-dimensional slices are taken per three-dimensional object, this creates many more samples to use for training a segmentation model, however, it comes at a cost of losing three-dimensional information about the cells in a sample. A three-dimensional model would potentially require significantly more data to train to the same accuracy as this work, but would have higher utility as three-dimensional metrics (e.g. surface area rather than perimeter) could be utilised to derive more information about the biology of IMPDH RRs.

### Dice/Jaccard variance

We see a particularly high variance in the segmentation metrics for the time course dataset, seemingly due to the interaction between sparse timepoints with few rods and rings and the calculation of the Dice and Jaccard scores (Additional File 4: Fig. S4). In cases where only a few pixels were annotated, small variations in the prediction compared with the ground truth have a stronger effect on these metrics. Moreover, if the only rod or ring structure is missed by the models (common in images with very few, hard to see rods or rings, of which none were in the training set), these metrics are extremely low. In images without rods or rings, the Dice and Jaccard scores both give errors (due to a zero denominator), so are excluded from the average metric calculation. However, since this corresponds to a biologically perfect segmentation, we have also included figures with these metrics included and set to 1 in Additional File 4: Fig. S4.

It should be noted that in these cases, the downstream analysis arguably provides a better picture of the model’s performance and utility in a realistic context. Low segmentation metrics do not necessarily lead to a biologically inaccurate model, as the model may still count the correct number of rings and measure the areas correctly with a slightly offset annotation.

### Measurement variance

**Fig. 9:**
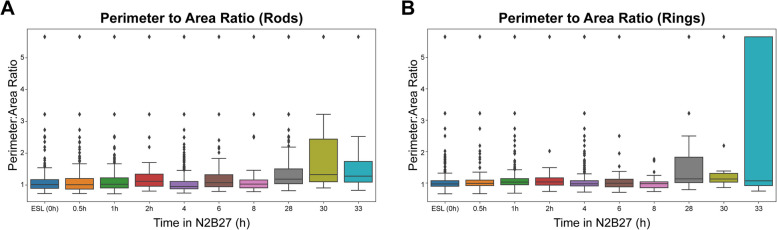
Perimeter-to-area ratio for the time course experiment. For each of the detected polygons, the perimeter and area were calculated to account for changes due to the change in media. However, due to the pixel size of the objects in the microscopy images, we see common ratios appear due to noisy segmentation. In future work, if this ratio is to be used, a higher resolution is needed for each of the objects to allow for more granularity in the perimeter and area calculations

Measuring correlation with expert annotations is dangerous for several reasons: firstly, it assumes that the expert annotation is a completely accurate source of truth (see below). Secondly, it is also weak to high variance measurements: for example, due to the resolution of the data, large changes in some measurements can occur when only small changes are made in the annotation. In this work, the model showed strong correlation between expert annotation and model output for rod and ring counts. This measurement shows lower variance when the sample size (i.e. the number of RRs available) is high. Since the microscope has a fixed resolution, this occurs when there are a large number of small, low-pixel count RRs available. Unfortunately, measuring the area or perimeter of the rings and rods can be difficult. When the RRs have a small number of pixels, an additional pixel labelled by the model results in a large change in the area and perimeter measurements. Additionally, the low resolution of these objects results in only a few possible values, leading to discontinuities in histograms when performing downstream analysis (Fig. 9). The solution to the above problem is to take images where fewer RRs are visible but at a much higher resolution, where the area and perimeters can be measured with a higher precision for analysis. However, this reduces the number of RRs visible, which itself increases variance for these measurements given the same number of images taken. When testing the effect of changing the microscope on the AI model performance, we see that the effective method to preserve model performance is to preprocess the data in a bespoke way that preserves the real-life height and width of a pixel in space. For other microscopes, this can be calculated easily but is crucial for model inference when using data generated from microscopes even when imaged at the same objective magnification as shown above.

### Expert bias

**Fig. 10:**
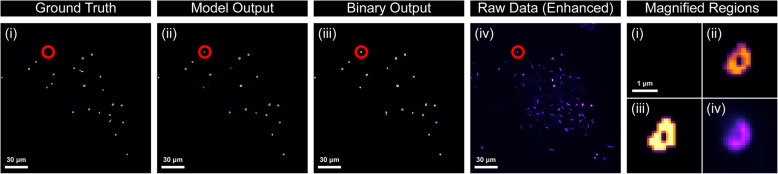
Comparison of model labelling rings potentially missed by experts. Examples of ground truth labelling performed by the microscopy expert (i), the model logit “fuzzy” output mask (ii), the binary mask for the images (iii), and the original image with increased exposure (iv). We see that a ring-like structure visible in the original image (circled in red, magnified to the far right) is picked up by the model but not annotated by the expert annotator. Scale bars represent 30 μm, or 1 μm for the magnified regions

In the recording of metrics for this work, we encounter a common problem in using AI for biological or medical tasks, which was the lack of an objective ground truth. While the results in this work are promising, these are metrics based on a comparison to expert annotation that may include biases and variances between and within the datasets that make annotations inconsistent. Some objects close to the edge of the image or objects slightly dimmed compared with other objects might be labelled as a ring or a rod by the expert but not by the AI model, or vice versa. This leads to some slight inconsistencies with the metrics, and it is arguable as to whether areas the model was marked as incorrect were incorrect at all (Fig. 10).

## Conclusions

We have developed an AI tool with an accompanying webapp to identify, classify, and quantitatively analyse IMPDH2 RR structures from confocal microscopy images. Furthermore, we have shown that our model works out of domain when images are taken on different microscopes, and when the size and conformation of RR structures differ (e.g. during differentiation).

The RR conformation is not unique to IMPDH2, nor are these structures limited to mammalian cells: depending on the biological context, different metabolic enzymes can form these structures, which can vary in size, frequency, and topology. Examples include CTPS in yeast [8, 31], IMPDH2 in zebrafish teeth [32], and various filamentous RR proteins in drosophila and yeast [8]. Additionally, other RR-like structures, distinct from both IMPDH2 RRs and CTPS1 cytoophidia, have been identified, such as nematosomes [33, 34] and loukoumasomes [15, 35], the latter enriched with cytoskeletal proteins. Therefore, the pipeline we have developed can be of broader significance to multiple groups, allowing them to train their own datasets and quantitatively analyse these structures.

Finally, biological systems do not exist in static form, and many proteins are highly dynamic. Indeed, temporal imaging of fluorescent CTPS1 and IMPDH2 has shown that RRs are dynamic structures, coalescing into rods and rings, and increasing/decreasing their size over time [7, 36]. Our model at present does not cater for time-lapse tracking of live RRs in real-time, but as segmentation and classification are usually the bottleneck, tracking segmented RRs could be a feasible modification for future pipeline developments.

## Supplementary Information

Additional File 1: Figure S1: Additional Metrics for model training. Each graph shows ground truth measurements on the x-axis vs the model’s ability to capture the average area, and average perimeter, perimeter/area for either rods or rings. The Rod:Ring ratio is also shown. *R*^2^ values are shown in each graph showing the correlation between the model’s ability to capture ground truth measurements.Comparison of Ground Truth Labels for training dataset with predictions, with particular errors highlighted. Black: True Positive; Blue: False Negative; Yellow: False Positive; Green: True Positive. Scale bar indicates 30 μm.

Additional File 2: Figure S2: Additional Metrics for the time-course. Each graph shows ground truth measurements on the x-axis vs the model’s ability to capture the average area, and average perimeter, perimeter/areafor either rods or rings. The Rod:Ring ratio is also shown. *R*^2^ values are shown in each graph showing the correlation between the model’s ability to capture ground truth measurements

Additional File 3: Figure S3: Additional metrics following modification of the microscopy pipeline. Each graph shows ground truth measurements on the x-axis vs the model’sability to capture the average area, and average perimeter, perimeter/areafor either rods or rings. The Rod:Ring ratio is also shown. *R*^2^ values are shown in each graph showing the correlation between the model’s ability to capture ground truth measurements

Additional File: Figure S4: Webapp developed for lab use. During this work, constant feedback was needed from microscopists for successful development of the mode. This WebApp was developed for use by microscopists to automatically segment the confocal microscopy image files and calculate the summary features from the segmentation. Code for self-hosting the web app, along for the code used to develop the models can be found here https://github.com/gastruloids/gandalf.

Additional File 5: Figure S5: Problematic Time course Dataset Annotations and Metric Analysis. In the time course dataset, some samples had fewor norod/ring structures. While the model output was reasonable in both these cases; due to the nature of the Dice and Jaccard scores, the metrics were very low for these samples. Since the model correctly segments the empty images, we also include another bar chart for the metricswith these errors manually set as a score of 1. We also include histograms for the different metricsfor the time course dataset to show that these corrections give artefacts in these histograms, with peaks at 1.0 where samples missing any rod/ring structures exist

## Data Availability

All software and code generated during this study are included in this published article’s additional information files and have been deposited in Zenodo https://doi.org/10.5281/zenodo.15268276 [30] and github [https://github.com/gastruloids/gandalf].

## Abbreviations

AI: Artificial intelligence
CTPS1: Cytidine triphosphate synthase 1
ESC: Embryonic stem cell
FBS: Foetal bovine serum
GMP/GTP: Guanine monophosphate/guanine triphosphate
IMPDH2: Inosine monophosphate dehydrogenase 2
PBS^−/−^: Phosphate buffered saline without calcium or magnesium ions
PBSFT: PBS^-/-^ supplemented with 10% FBS and 0.2% Triton X100
PBT: PBS^-/-^supplemented with 0.2% FBS and 0.2% Triton X100
RR: Rod and ring
