# Supplementary figures and images for: Domain-specific AI segmentation of IMPDH2 rod/ring structures in mouse embryonic stem cells
Source: BMC Biol. 2025 May 12;23:126. doi: 10.1186/s12915-025-02226-7 (PMC12067766; doi:10.1186/s12915-025-02226-7)

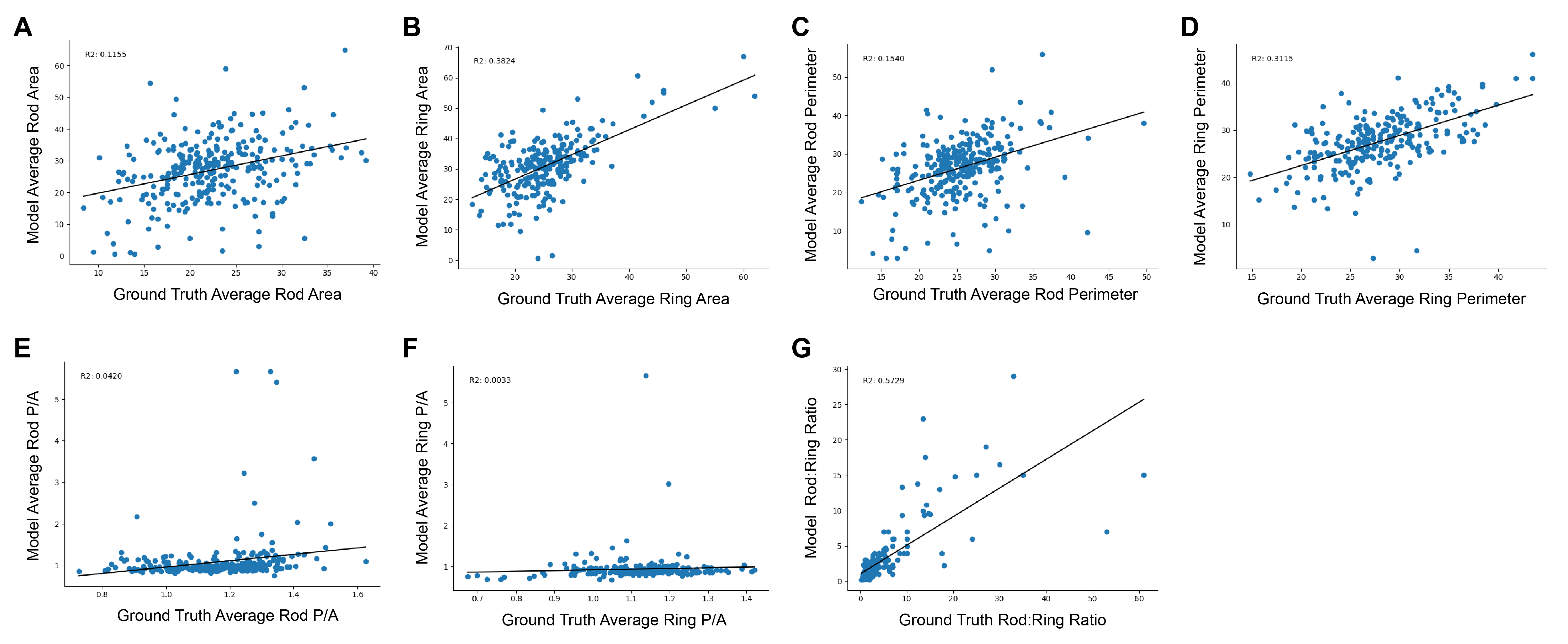

Supplement: Supplementary file 2 — Additional File 2: Figure S2: Additional Metrics for the time-course. Each graph shows ground truth measurements on the x-axis vs the model’s ability to capture the average area, and average perimeter, perimeter/areafor either rods or rings. The Rod:Ring ratio is also shown. R2 values are shown in each graph showing the correlation between the model’s ability to capture ground truth measurements [file 12915_2025_2226_MOESM2_ESM.png]

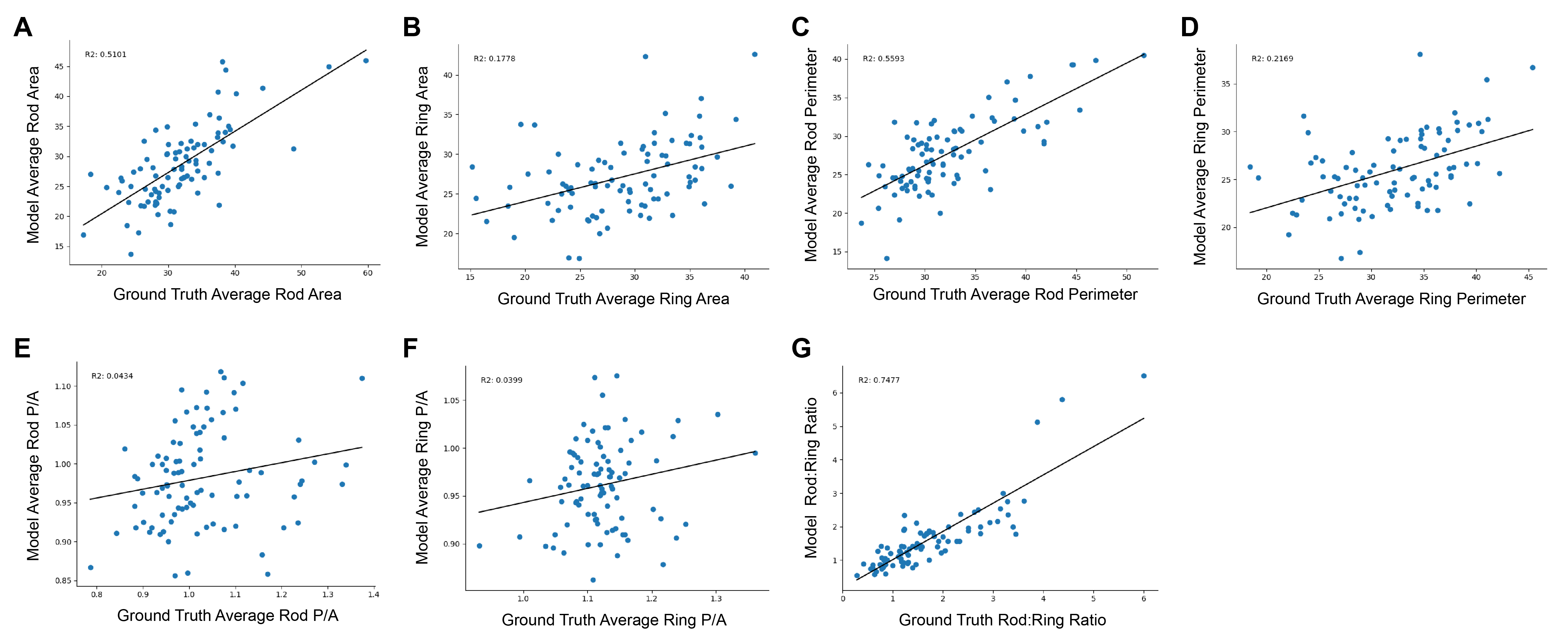

Supplement: Supplementary file 3 — Additional File 3: Figure S3: Additional metrics following modification of the microscopy pipeline. Each graph shows ground truth measurements on the x-axis vs the model’sability to capture the average area, and average perimeter, perimeter/areafor either rods or rings. The Rod:Ring ratio is also shown. R2 values are shown in each graph showing the correlation between the model’s ability to capture ground truth measurements [file 12915_2025_2226_MOESM3_ESM.png]

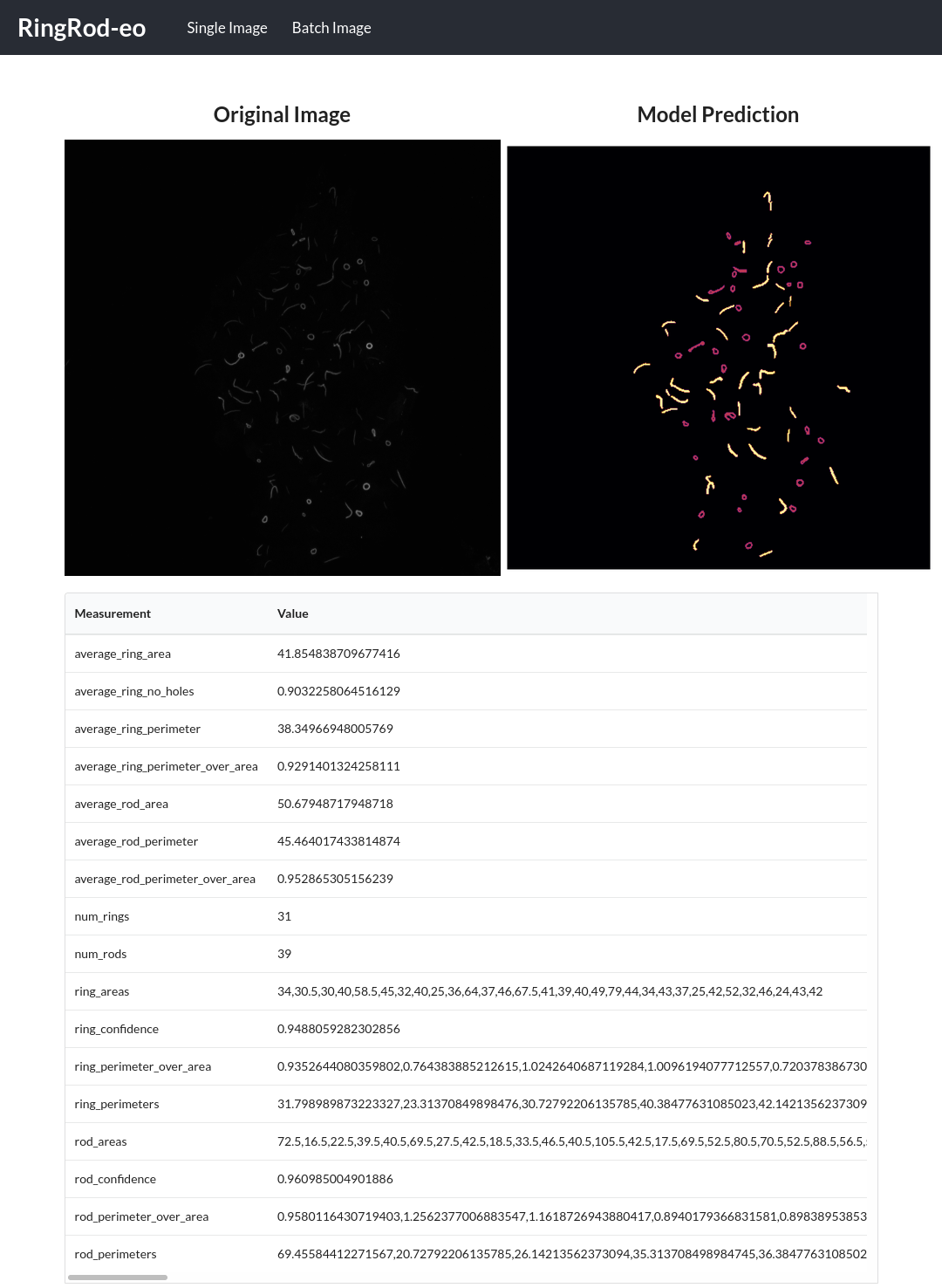

Supplement: Supplementary file 4 — Additional File: Figure S4: Webapp developed for lab use. During this work, constant feedback was needed from microscopists for successful development of the mode. This WebApp was developed for use by microscopists to automatically segment the confocal microscopy image files and calculate the summary features from the segmentation. Code for self-hosting the web app, along for the code used to develop the models can be found here https://github.com/gastruloids/gandalf. [file 12915_2025_2226_MOESM4_ESM.png]

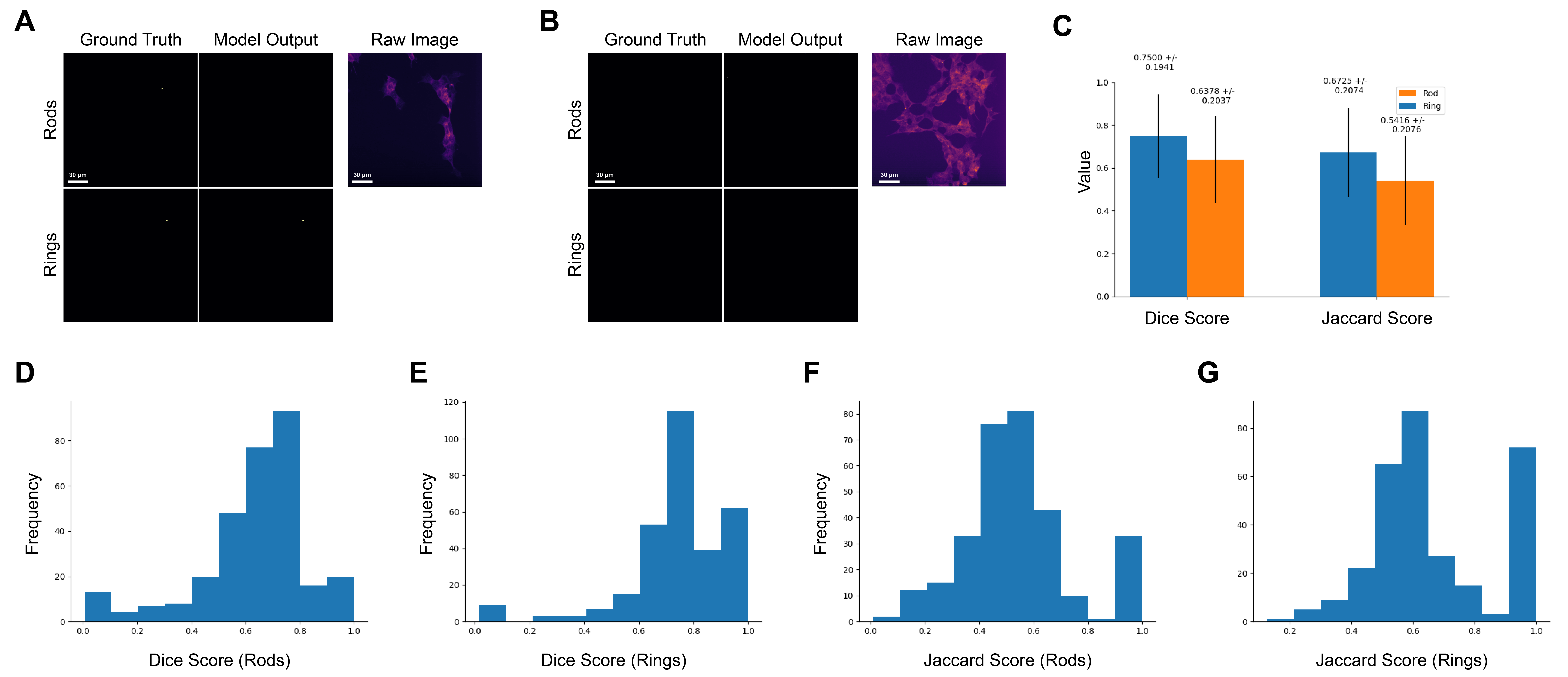

Supplement: Supplementary file 5 — Additional File 5: Figure S5: Problematic Time course Dataset Annotations and Metric Analysis. In the time course dataset, some samples had fewor norod/ring structures. While the model output was reasonable in both these cases; due to the nature of the Dice and Jaccard scores, the metrics were very low for these samples. Since the model correctly segments the empty images, we also include another bar chart for the metricswith these errors manually set as a score of 1. We also include histograms for the different metricsfor the time course dataset to show that these corrections give artefacts in these histograms, with peaks at 1.0 where samples missing any rod/ring structures exist [file 12915_2025_2226_MOESM5_ESM.png]
